# Integrated healthcare approach can curb the increasing cases of cryptococcosis in Africa

**DOI:** 10.1371/journal.pntd.0010625

**Published:** 2022-08-25

**Authors:** Chibuike Ibe, Chinonyelum Annette Okoye

**Affiliations:** Department of Microbiology, Faculty of Biological Sciences, Abia State University, Uturu, Nigeria; Rutgers University, UNITED STATES

## Abstract

Cryptococcosis is a neglected tropical infection and a major cause of morbidity and mortality, especially in HIV–positive persons in Africa. Efforts to manage HIV infection have not had any significant impact on the fatalities due to cryptococcosis. An integrated healthcare approach that includes universal care coverage for Africans, expanded national care guidelines to include CrAg screening for vulnerable groups in all African countries, collaborative research, infection surveillance, and data sharing within Africa will mark a turnaround point.

*Cryptococcus neoformans* and *Cryptococcus gattii* are environmental saprophytes found in the soil, pigeon, and poultry droppings. They are the commonest cause of cryptococcosis characterised with mild superficial or asymptomatic but self-limiting infections in healthy individuals to severe life-threatening invasive infections in immune-impaired persons especially in those living with HIV infection. The infection begins with the inhalation of infectious propagules from an environmental reservoir into the pulmonary alveoli leading to various clinical manifestations based on the specific characteristics of patient population and their immune status. The most common manifestation of cryptococcosis and the major cause of deaths is meningoencephalitis. About a million cases of cryptococcosis occur annually resulting in more than 625,000 deaths with more than 70% of these cases and deaths occurring in sub-Saharan Africa [1].

Cryptococcosis became common in the 1980s following the discovery of HIV, showing a correlation between high prevalence of HIV infection with increasing cryptococcosis spread and fatalities. The introduction of antiretroviral therapy (ART) was thought to have a drastic negative effect on the spread of cryptococcosis. However, the reality is that even with the successful coverage of ART in sub-Saharan Africa, cases and fatalities due to cryptococcosis are still on the rise (Fig 1) [2]. The coverage of ART has been affected by defaulters, so many naïve persons who are unaware of their HIV infection and ART failure/viral resistance, all of which contribute positively to cryptococcosis spread. Additionally, the cost and unavailability of reliable diagnostics, limited availability of effective therapeutic options, and immune-crippling comorbidities such as tuberculosis and pneumocystis pneumonia can complicate cryptococcosis patients’ conditions and significantly affect outcome.

**Fig 1:**
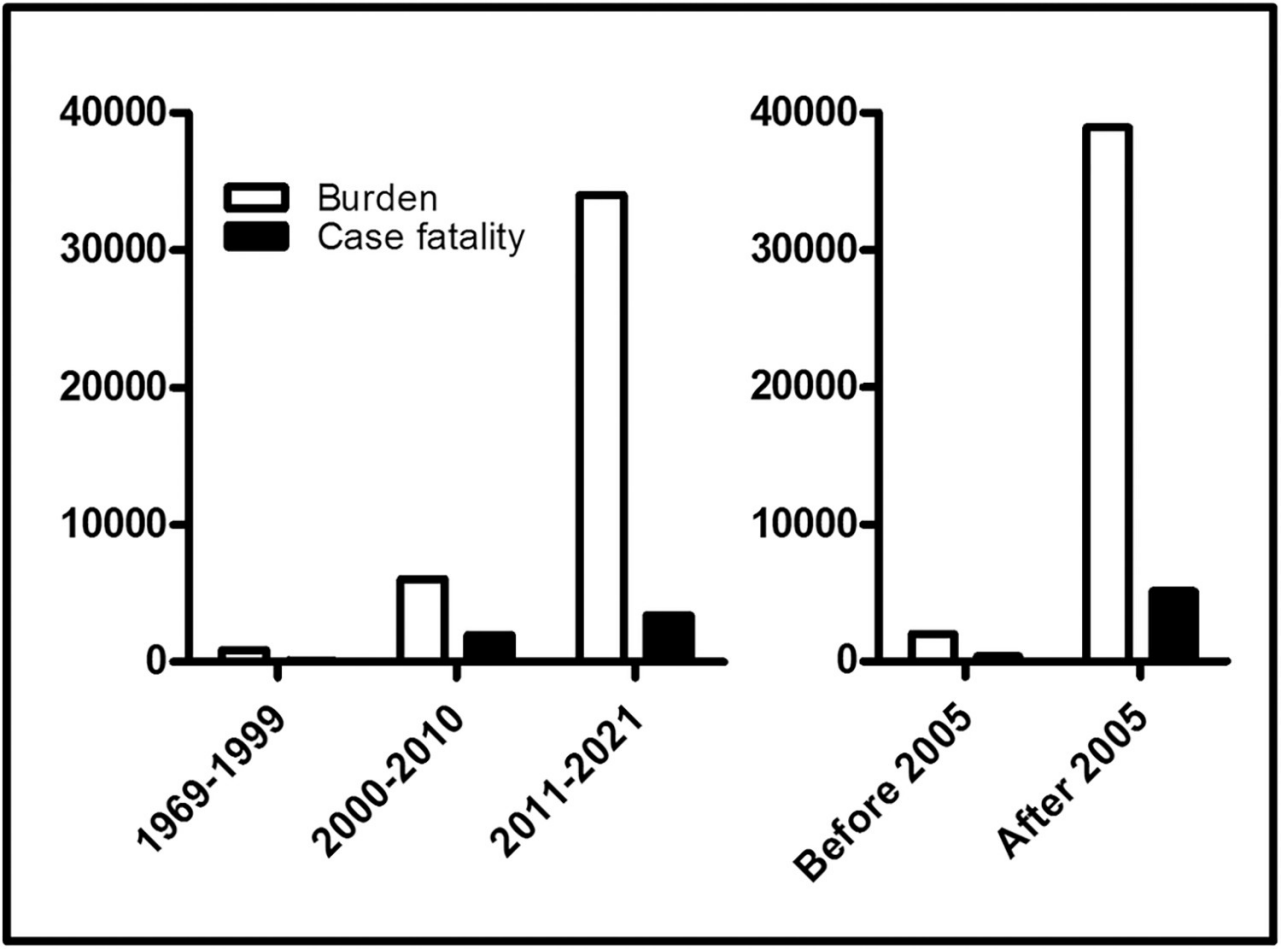
Data from 209 studies showing cryptococcosis burden and case fatalities in decades before and after HIV discovery (left) and burden and fatalities before and after wide ART coverage (right) in Africa. Following the discovery of HIV, cryptococcosis has been on a steady increase in Africa including deaths due the infection. It is possible that the increasing cases were due to the attention drawn to the infection leading to more studies and large-scale retrospective and prospective studies conducted over time especially in the last decade and since there is rising incidence of HIV infection in Africa. The cost of diagnosing cryptococcosis and the unavailability of effective antifungal therapies in Africa are also factors that have contributed to the increasing case fatalities. Data showed that in 2011–2021, cryptococcosis is more in HIV–negative persons (18,047 compared to 15,956 HIV–positive persons; see Tables A and B in S1 File) that may largely be because some studies conducted in South Africa and Botswana did not report the HIV status of their participants (left). From available data, 2005 was marked as a turnaround point for ART coverage and hold that more countries in Africa at this point may have reached at least 60%–70% in the first wave of ART coverage. Acknowledging the possible overlap in the marked point of 2005 and the increase in research interest in cryptococcosis and attention from WHO, GAFFI, LIFE, and CDC/CHAI/Unitaid global initiatives in Africa may partly explain the increase in the burden of cryptococcosis after the marked wide ART coverage point. However, with the relative availability of CrAg test and the moderately effective fluconazole and amphotericin B deoxycholate that are widely available in Africa, it is still evident that the successful coverage of ART has not had any meaningful effect on the prevalence of cryptococcosis and deaths due to the infection in Africa (right). An integrated healthcare approach may provide the right effect required to have a turnaround point.

Sub-Saharan Africa is struggling with the highest share of HIV infection, cryptococcosis has been a major problem in HIV–positive persons, and it was estimated to account for more than 50% of fungal-derived HIV complications [1] especially in those with CD4 lymphocyte ≤100 cells/μl that has remained relatively the same over time [3]. Flucytosine and liposomal amphotericin B combination therapy is the best of drug choice for cryptococcosis [4], but they are very expensive and only available in Tanzania, Morocco, Zimbabwe, South Africa, Malawi, Eswatini, and Ghana; Benin, Egypt, Ethiopia, Tanzania, Mauritania, Eswatini, and South Africa, respectively. Treatment of cryptococcosis in Africa is mainly with fluconazole and amphotericin B deoxycholate that only increases patients’ survival by about 30% while in the absence of treatment fatality is almost 100% [4]. There are now reports of widespread resistance to fluconazole and to a lesser degree amphotericin B deoxycholate that has significantly affected treatment outcome.

Gross domestic product (GDP) has a significant negative relationship with cryptococcosis infections and fatalities. Countries with lower GDP have higher prevalence of cryptococcosis [5]. High GDP translates to developed economy and more advanced healthcare system with high density of healthcare workforce. The availability of healthcare facilities and well-trained medical workforce to provide supportive care is a challenge in sub-Saharan African where doctors, nurses, and laboratory scientists with training in mycology are scarce (see Table A in S2 File). African countries have the lowest density of nurses and medical doctors per 10,000 population; there are about 13.4 nurses and 3.3 medical doctors (see Table A in S2 File). This is severe in some countries including Malawi that has around 4.4 nurses and 0.4 medical doctors per 10,000 of its population (see Table A in S2 File) [5]. In Europe, however, there are 77.8 nurses and 43.2 medical doctors per 10,000 population, the United Kingdom, for example, has 102.9 nurses and 58.2 medical doctors. This can explain why cryptococcosis is an infection of poor resource regions of the world partly due to low health expenditure (see Table A in S2 File). Most African countries have poorly funded, insufficient, and overburdened healthcare and public health systems that cannot cope with pressure from the infection and the complex medical care required by patients with cryptococcal meningoencephalitis who may also have reduced consciousness. Optimum medical care for meningoencephalitis includes low-risk specimen collection skills such as for lumbar puncture, standard laboratory tests, intravenous drug administration, and observation and tests for neurological complications and drug toxicity. This is besides screening and management of other comorbidities.

Cryptococcosis unspecific and complex clinical manifestations require a robust healthcare management approach. Managing this complex medical requirement for cryptococcosis patients could be daunting for any country and even more so for sub-Saharan African countries such as Nigeria that a recent WHO survey ranked its healthcare system as the fourth worst in the world. Compared to other West African countries with similar income, Nigeria’s healthcare outcomes are dismal, with significant variations between the rural and urban communities, poor and rich populations, and different regions [6] where infectious and communicable infections are common.

Cryptococcosis cases and deaths are underreported and underrepresented in sub-Saharan Africa because of limited infection surveillance, public health records, and published data. It is currently believed that HIV–positive persons especially those with advanced condition or CD4 lymphocyte ≤100 cells/μl are more susceptible to cryptococcosis complications-derived deaths than tuberculosis, [1,4] and the infection remains neglected in research, funding, and policy. Available data are rather more on cryptococcosis prevalence, drug susceptibility, or case report studies. There is no active research on the molecular and cellular mechanisms underlying the pathogenesis and pathology of cryptococcosis taking place in Africa. In the pathology of meningoencephalitis, the brain immune cells are important in deciding the progress of the infection and survival of a patient, yet no study in Africa has shown any critical understanding of the interplay between host adaptive immune responses and *Cryptococcus* species [7]. Immune-induced susceptibility to cryptococcosis in HIV–positive persons in Africa is poorly understood. This has significantly affected our understanding of the infection manifestation in this population in Africa.

There is no active cryptococcosis research group in Africa with optimal manpower and sufficient grant funding and this has affected research output. It has been argued that limited medical mycology research funding reflects limited grant application from the few researchers in the field. However, research funding is generally limited in Africa. In Nigeria, the only consistent funding body for life sciences research is tertiary education trust fund (TETFund) institution-based research (IBR) and national research fund (NRF) grants. TETFund grant has a broad scope and may insufficiently fund certain “high-priority” areas. In addition, the terms of funding research are narrow. The limited research or funding for research has created gaps in our efforts to tackle the continued spread of cryptococcosis in Africa. For example, *C*. *neoformans* is the most common cause of cryptococcosis in Africa; however, an epidemic shift to *C*. *gattii* (serotype C) is suspected soon [8]. *C*. *gattii* (serotype C) causes more severe infections in advanced HIV–positive patients, does not respond well to antifungal treatment, and much is not known yet about its biology. Recent study in Malawi and Botswana has shown that *C*. *gattii* (serotype C) is responsible for the large proportion of cryptococcosis in HIV–positive persons that could partly explain the high burden of the infection in Africa [8]. C. *gattii* (serotype C) poses a greater threat to African health than is known and has attracted little or no attention in research.

The havoc cryptococcosis has wreaked on the African continent calls for an integrated healthcare approach especially for HIV–positive persons with CD4 lymphocytes count ≤100 cells/μl to include CrAg screening into the national healthcare system guideline of all countries in Africa. Screening HIV–positive persons for cryptococcosis is needed for early diagnosis and effective antifungal treatment to prevent progression of infection and has been shown to provide survival benefits [9]. So far, only few countries including Kenya, Swaziland, Mozambique, Namibia, Zimbabwe, Rwanda, Botswana, South Africa, and Uganda have incorporated CrAg screening as part of their national healthcare guideline for HIV infection. In Uganda, for example, CrAg screening and preemptive treatment programmes have been shown to be both life and cost saving assuming treatment is more than 75% effective [10]. This programme can easily be taken by the ministry of health of any country in Africa and implemented to reduce cryptococcosis mortality in HIV–positive persons. HIV specialised/differential healthcare model must be developed such as the function of the “CrAg screening champion” [11] that can help patients keep to ART regimen and minimise defaulting and treatment failures while promoting viral load suppression first at an individual level. In Uganda, targeted care structure has proved effective in ensuring patients’ compliance [11].

Integrated healthcare investment [12] and healthcare policy reforms will promote good health outcome and socioeconomic development. For example, the Nigerian government through TETFund has invested huge funds in training healthcare workers and academics to acquire basic and advanced training in their chosen area (www.tetfund.gov.ng). This will over time increase the density of healthcare workforce to support the provision of a healthcare plan such as universal healthcare coverage that can benefit the middle- and lower-income class and prevent catastrophic out-of-pocket payment that is the common means of covering health expenses by the poor populace in Africa. Increased health spending in South Africa has increased healthcare workforce to support their integrated and unified health systems that reduced out-of-pocket spending significantly compared to Nigeria (Table A in S2 File). Incorporation of cryptococcosis screening as part of doctors’ risk assessment to rule out the infection in those with other severe vulnerabilities and at-risk groups and antifungal therapy resumed in positive persons to prevent fatal manifestation would also improve health outcomes. Integrated healthcare approach will also involve collaborative research and capacity building involving multidisciplinary, multisectoral, and innovative strategies, knowing the inseparable nature of humans from their environment, to tackle the problem of cryptococcosis and associated complications. Greater interaction between health sectors of African countries in sharing their successes and failures in cryptococcosis responses is important.

Adopting one health approach and government involvement in instituting more research funding bodies in partnership with the private sector to drive research on understanding the molecular basis and immunological responses and *Cryptococcus* countermeasures that define infection manifestation in Africa carry great potential. Knowledge of *Cryptococcus* species can help develop rapid and accurate diagnostics and effective and less toxic therapeutics for managing cryptococcosis. More research funding bodies will drive multisectoral infection surveillance, laboratory involvement, public health record keeping, and increase societal awareness which are crucial to promote the importance of good community and public health practices. In Uganda, lack of knowledge of the infection has been shown to affect cryptococcosis diagnosis and care [13]. Research on understanding the immunologically driven susceptibility to cryptococcosis in HIV–positive persons can help develop immunotherapeutic strategies for managing cryptococcosis in those with immune-crippling infections in Africa [14]. Integrated healthcare approach might just be the right strategy to urgently and holistically tackle cryptococcosis and its associated complications in Africa and elsewhere.

## Supporting information

S1 File Table A. Burden and fatalities from cryptococcosis before and after HIV discovery. Table B. Burden and fatalities from cryptococcosis before and after the wide coverage of antiretroviral therapy.
(DOCX)

S2 File Table A. Population size and health investment characteristics of African countries.
(DOCX)
