# Supplementary material for: Integrated healthcare approach can curb the increasing cases of cryptococcosis in Africa
Source: PLoS Negl Trop Dis. 2022 Aug 25;16(8):e0010625. doi: 10.1371/journal.pntd.0010625 (PMC9409514; doi:10.1371/journal.pntd.0010625)
Supplement: S1 File — Table A. Burden and fatalities from cryptococcosis before and after HIV discovery. Table B. Burden and fatalities from cryptococcosis before and after the wide coverage of antiretroviral therapy. (DOCX) [file pntd.0010625.s001.docx]

Supplementary information

Table A: Burden and fatalities from cryptococcosis before and after HIV discovery

|  | Burden | HIV +ve | HIV -ve | Case fatality |
| --- | --- | --- | --- | --- |
| 1969-1999 | 895 | 496 | 399 | 118(0.13) |
| 2000-2010 | 6050 | 4011 | 2039 | 1974(0.33) |
| 2011-2021 | 34003 | 15956 | 18047 | 3408(0.1) |

HIV +ve = HIV positive people, HIV -ve = HIV negative people, ART, antiretroviral therapy

Table B: Burden and fatalities from cryptococcosis before and after the wide coverage of antiretroviral therapy

|  | Burden | HIV +ve | HIV -ve | Case fatality |
| --- | --- | --- | --- | --- |
| Before ART wide coverage (before 2005) | 2017 | 1332 | 685 | 388(0.2) |
| After ART wide coverage (after 2005) | 38931 | 19131 | 19800 | 5112(0.13) |

HIV +ve = HIV positive people, HIV -ve = HIV negative people, ART, antiretroviral therapy

**Case definition**

In this draft, the term cryptococcosis was used to denote all cases of cryptococcal infection in any part of the body including pulmonary and meninges cryptococcosis caused by any species of *Cryptococcus*. Cases of cryptococcosis included in this review are all cases confirmed by direct microscopic examination, cytopathology or histological examination of biopsy or needle aspiration; culture of blood or cerebrospinal fluid, or other samples collected from sterile sites; cryptococcal antigen (CrAg) test of blood or cerebrospinal fluid; or polymerase chain reaction (PCR) amplification of cryptococcal DNA ^1^.

Reference

1. Peter Donnelly, J. *et al.* Revision and update of the consensus definitions of invasive fungal disease from the European organization for research and treatment of cancer and the mycoses study group education and research consortium. *Clinical Infectious Diseases* **71**, 1367–1376 (2020).
