# Supplementary material for: Integrated healthcare approach can curb the increasing cases of cryptococcosis in Africa
Source: PLoS Negl Trop Dis. 2022 Aug 25;16(8):e0010625. doi: 10.1371/journal.pntd.0010625 (PMC9409514; doi:10.1371/journal.pntd.0010625)
Supplement: S2 File — Table A. Population size and health investment characteristics of African countries. (DOCX) [file pntd.0010625.s002.docx]

Supplementary information

Table A: Population size and health investment characteristics of African countries

| **Country** | **Population size^1^** | **Health expenditure (% GDP) (2019)^3^** | **Out-of-pocket expenditure (% health expenditure) (2019)^3^** | **Density per 10 000 population (2011-2019)^2^** | | **Population that needs interventions against NTDs (2019)^2^** |
| --- | --- | --- | --- | --- | --- | --- |
|  |  |  |  | **Nurse** | **Doctor** |  |
| Nigeria | 206 139 589 | 3.0 | 70.5 | 15.0 | 3.8 | 134 545 208 |
| Ethiopia | 114 963 588 | 3.2 | 37.8 | 7.1 | 0.8 | 76 238 251 |
| Egypt | 102 334 404 | 4.7 | 62.7 | 19.3 | 7.5 | 6 894 411 |
| DR Congo | 89 561 403 | 3.5 | 38.5 | 11.1 | 0.9 | 53 320 501 |
| Tanzania | 59 734 218 | 3.8 | 22.1 | 5.8 | 0.6 | 27 086 592 |
| South Africa | 59 308 690 | 9.1 | 5.6 | 13.1 | 7.9 | 18 807 465 |
| Kenya | 53 771 296 | 4.5 | 24.3 | 11.7 | 1.6 | 8 321 398 |
| Uganda | 45 741 007 | 3.8 | 38.2 | 12.4 | 1.7 | 24 639 995 |
| Sudan | 43 849 260 | 4.5 | 67.3 | 11.5 | 2.6 | 12 015 065 |
| Algeria | 43 851 044 | 6.2 | 33.4 | 15.5 | 17.2 | 10 339 |
| Morocco | 36 910 560 | 5.3 | 46.8 | 13.9 | 7.3 | 5 576 |
| Angola | 32 866 272 | 2.5 | 37.4 | 4.1 | 2.1 | 15 362 008 |
| [Mozambique](https://worldpopulationreview.com/countries/mozambique-population) | 31 255 435 | 7.8 | 9.9 | 4.7 | 0.8 | 21 517 399 |
| [Ghana](https://worldpopulationreview.com/countries/ghana-population) | 31 072 940 | 3.4 | 36.2 | 27.1 | 1.1 | 17 220 101 |
| [Madagascar](https://worldpopulationreview.com/countries/madagascar-population) | 27 691 018 | 3.6 | 32.5 | 3.0 | 1.8 | 21 125 732 |
| [Cameroon](https://worldpopulationreview.com/countries/cameroon-population) | 26 545 863 | 3.6 | 72.5 | 0.1 | 0.9 | 16 891 418 |
| [Ivory Coast](https://worldpopulationreview.com/countries/ivory-coast-population) | 26 378 274 | 3.3 | 37.2 | 6.6 | 1.6 | 21 713 670 |
| [Niger](https://worldpopulationreview.com/countries/niger-population) | 25 847 386 | 5.6 | 46.1 | 2.2 | 0.4 | 14 046 246 |
| [Burkina Faso](https://worldpopulationreview.com/countries/burkina-faso-population) | 20 903 273 | 5.4 | 34.6 | 9.3 | 0.9 | 3 652 080 |
| [Mali](https://worldpopulationreview.com/countries/mali-population) | 20 250 833 | 3.8 | 31.3 | 4.4 | 1.3 | 7 735 946 |
| [Malawi](https://worldpopulationreview.com/countries/malawi-population) | 19 129 952 | 7.3 | 16.8 | 4.4 | 0.4 | 12 393 153 |
| [Zambia](https://worldpopulationreview.com/countries/zambia-population) | 18 383 955 | 5.3 | 10.2 | 10.2 | 0.9 | 12 032 435 |
| [Senegal](https://worldpopulationreview.com/countries/senegal-population) | 16 743 927 | 4.1 | 50.9 | 5.4 | 0.9 | 8 815 619 |
| [Chad](https://worldpopulationreview.com/countries/chad-population) | 16 425 864 | 4.3 | 57.2 | 1.4 | 0.5 | 6 270 047 |
| [Somalia](https://worldpopulationreview.com/countries/somalia-population) | 15 893 222 | - | - | 1.1 | 0.2 | 2 286 299 |
| [Zimbabwe](https://worldpopulationreview.com/countries/zimbabwe-population) | 14 862 924 | 7.7 | 24.3 | 19.3 | 2.1 | 10 660 813 |
| [Guinea](https://worldpopulationreview.com/countries/guinea-population) | 13 132 795 | 3.9 | 59.2 | 1.2 | 0.8 | 7 480 197 |
| [Rwanda](https://worldpopulationreview.com/countries/rwanda-population) | 12 952 218 | 6.4 | 11.6 | 9.5 | 1.2 | 5 015 979 |
| [Benin](https://worldpopulationreview.com/countries/benin-population) | 12 123 200 | 2.3 | 47.0 | 3.0 | 0.6 | 6 070 676 |
| [Burundi](https://worldpopulationreview.com/countries/burundi-population) | 11 890 784 | 7.9 | 24.6 | 6.6 | 1.0 | 3 418 124 |
| [Tunisia](https://worldpopulationreview.com/countries/tunisia-population) | 11 818 619 | 6.9 | 37.9 | 25.1 | 13.0 | 7 085 |
| [South Sudan](https://worldpopulationreview.com/countries/south-sudan-population) | 11 193 725 | 6.0 | 23.5 | - | - | 8 472 338 |
| [Togo](https://worldpopulationreview.com/countries/togo-population) | 8 278 724 | 5.7 | 66.2 | 4.6 | 0.8 | 4 311 460 |
| [Sierra Leone](https://worldpopulationreview.com/countries/sierra-leone-population) | 7 976 983 | 8.7 | 55.1 | 7.5 | 0.7 | 6 910 107 |
| [Libya](https://worldpopulationreview.com/countries/libya-population) | 6 871 292 | 6.0 | 36.6 | 65.3 | 20.9 | 6 774 |
| Congo | 5 518 087 | 2.0 | 45.8 | 9.3 | 1.1 | 1 407 153 |
| [Liberia](https://worldpopulationreview.com/countries/liberia-population) | 5 057 681 | 8.4 | 54.4 | 5.3 | 0.4 | 3 175 460 |
| [Central African Republic](https://worldpopulationreview.com/countries/central-african-republic-population) | 4 829 767 | 7.7 | 60.3 | 2.1 | 0.7 | 4 442 825 |
| [Mauritania](https://worldpopulationreview.com/countries/mauritania-population) | 4 649 658 | 3.3 | 45.0 | 9.3 | 1.9 | 826 827 |
| [Eritrea](https://worldpopulationreview.com/countries/eritrea-population) | 3 546 421 | 4.4 | 43.2 | 14.4 | - | 427 112 |
| [Namibia](https://worldpopulationreview.com/countries/namibia-population) | 2 540 905 | 8.5 | 8.2 | 19.5 | 5.9 | 1 094 020 |
| [Gambia](https://worldpopulationreview.com/countries/gambia-population) | 2 416 668 | 3.8 | 23.1 | 6.1 | 1.1 | 168 211 |
| [Botswana](https://worldpopulationreview.com/countries/botswana-population) | 2 351 627 | 6.0 | 3.1 | 54.6 | 2.9 | 238 203 |
| [Gabon](https://worldpopulationreview.com/countries/gabon-population) | 2 225 734 | 2.7 | 23.0 | 29.5 | 6.8 | 937 923 |
| [Lesotho](https://worldpopulationreview.com/countries/lesotho-population) | 2 142 249 | 11.2 | 13.7 | 32.6 | - | 382 336 |
| [Guinea Bissau](https://worldpopulationreview.com/countries/guinea--bissau-population) | 1 968 001 | 8.3 | 65.1 | 6.9 | 1.3 | 1 232 549 |
| [Equatorial Guinea](https://worldpopulationreview.com/countries/equatorial-guinea-population) | 1 402 985 | 3.1 | 75.4 | 5.0 | 4.0 | 429 326 |
| [Mauritius](https://worldpopulationreview.com/countries/mauritius-population) | 1 271 768 | 6.2 | 42.7 | 35.2 | 25.3 | 0 |
| [Eswatini](https://worldpopulationreview.com/countries/eswatini-population) | 1 160 164 | 6.7 | 10.5 | 41.4 | 1,0 | 406 184 |
| [Djibouti](https://worldpopulationreview.com/countries/djibouti-population) | 988 000 | 1.8 | - | 2.2 | 7.3 | 110 561 |
| Comoros | 869,601 | 5.1 | 61.8 | 6.3 | 1.7 | 788 813 |
| [Cape Verde](https://worldpopulationreview.com/countries/cape-verde-population) | 555 987 | 4.8 | 24.9 | 13.0 | 7.8 | 137 073 |
| [Sao Tome and Principe](https://worldpopulationreview.com/countries/sao-tome-and-principe-population) | 219 159 | 5.5 | 17.6 | 3.2 | 19.2 | 201 114 |
| [Seychelles](https://worldpopulationreview.com/countries/seychelles-population) | 98 347 | 5.1 | 25.2 | 98.5 | 24.7 | 0 |
|  | **North sum = 245 635 179**  **Sub-Sahara sum = 1 079 950 945**  **Total sum = 1 342 467 346** | **North mean = 5.6**  **Sub-Sahara mean = 5.3**  **Total mean = 5.2** | **North mean = 47.5**  **Sub-Sahara mean = 36.2**  **Total mean = 37.5** | **North mean = 25.1**  **Sub-Sahara mean = 13.4**  **Total mean = 14.3** | **North mean = 11.4**  **Sub-Sahara mean = 3.3**  **Total mean = 4.3** | **North sum = 18 939 250**  **Sub-Sahara sum = 590 370 087**  **Total sum = 611 706 197** |

GDP, gross domestic product; NTDs, neglected tropical diseases; yr, year

^1^ www.worldpopulationreview.com

^2^www.who.int

^3^www.worldbank.org
